# Dissociable rhythmic mechanisms enhance memory for conscious and nonconscious perceptual contents

**DOI:** 10.1073/pnas.2211147119

**Published:** 2022-10-27

**Authors:** Phillip (Xin) Cheng, Shrey Grover, Wen Wen, Shruthi Sankaranarayanan, Sierra Davies, Justine Fragetta, David Soto, Robert M. G. Reinhart

**Affiliations:** ^a^Department of Psychological and Brain Sciences, Boston University, Boston, MA 02215;; ^b^Department of Biomedical Engineering, Boston University, Boston, MA 02215;; ^c^Basque Center on Cognition, Brain and Language, 20009 San Sebastian, Spain;; ^d^Ikerbasque, Basque Foundation for Science, 48009 Bilbao, Spain;; ^e^Center for Systems Neuroscience, Boston University, Boston, MA 02215;; ^f^Cognitive Neuroimaging Center, Boston University, Boston, MA 02215;; ^g^Center for Research in Sensory Communication and Emerging Neural Technology, Boston University, Boston, MA 02215

**Keywords:** high-definition transcranial alternating current stimulation, conscious awareness, unconscious processing, short-term memory, neural rhythms

## Abstract

Understanding the neural mechanisms of conscious and unconscious experience is a major goal of fundamental and translational neuroscience. Here, we target the early visual cortex with a protocol of noninvasive, high-resolution alternating current stimulation while participants performed a delayed target–probe discrimination task and reveal dissociable mechanisms of mnemonic processing for conscious and unconscious perceptual contents. Entraining β-rhythms in bilateral visual areas preferentially enhanced short-term memory for seen information, whereas α-entrainment in the same region preferentially enhanced short-term memory for unseen information. The short-term memory improvements were frequency-specific and long-lasting. The results add a mechanistic foundation to existing theories of consciousness, call for revisions to these theories, and contribute to the development of nonpharmacological therapeutics for improving visual cortical processing.

Determining the human brain mechanisms supporting conscious and unconscious information processing remains one of the most challenging endeavors in psychology and neuroscience (1; see ref. 2 for a recent review of theories of consciousness).* Rhythmic neural activity derived from postsynaptic currents is fundamental to information processing (3), and a major mechanism under study in consciousness research (4). For example, long-range β- and γ-synchronization (5) and α-desynchronization (6) have been proposed as the substrate of conscious access. However, previous findings on the rhythmic basis of consciousness have been correlational (7, 8). It remains unclear whether specific and dissociable neural rhythms causally drive conscious and unconscious information processing. Although theories emphasize the difference in the spatial scale of rhythms (global versus local) involved in conscious versus unconscious processing (e.g., ref. 1), other rhythmic dimensions can also be distinct. One dimension is frequency, but few studies in the neuroscience of consciousness have addressed it (9). In particular, it is unclear whether specific frequency bands selectively modulate task-relevant information depending on access to consciousness.

Here, we developed a high-definition (2 × 4) transcranial alternating current stimulation (HD-tACS) protocol to entrain visual cortical activity with maximal anatomical precision while participants performed a visual short-term memory task involving delayed target–probe discrimination decisions, wherein the target was masked from visual awareness (Fig. 1*A*). The rhythmic frequency for processing visual information should depend on conscious access. Specifically, since β-rhythms have been associated with conscious visual processing (10), entraining β-rhythms should improve the target–probe discrimination when the target is seen. In contrast, research has not tied a certain frequency band of neural rhythms to processing unconscious information. Some theories propose a rhythm-silent processing applicable to unconscious information (1, 11). However, neural rhythms might still contribute to unconscious information processing in a frequency-specific manner. If this is true, then entraining rhythms in this frequency should influence the target–probe discrimination when the target is unseen.

**Fig. 1.**
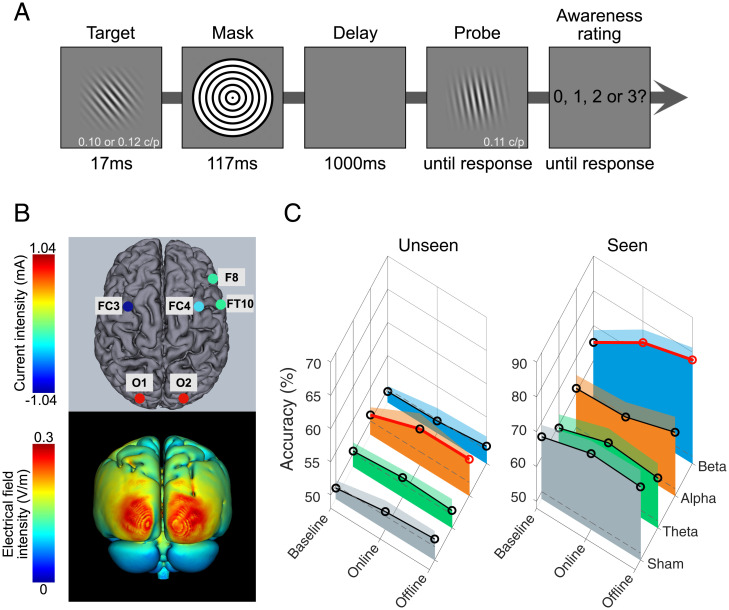
Task, neuromodulation protocol, and results. Complete results are available in OSF Results (https://osf.io/fqxk9/). (*A*) On each trial, a target stimulus (contrast = 0, 0.25, 0.75, or 1 with equiprobability) with an unpredictable orientation appeared for 17 ms, masked by radial square waves. After a 1,000-ms delay, a probe stimulus appeared. The participants’ task was to indicate, via a keypress, whether the target needed to be rotated clockwise or counter-clockwise to match the probe. They then rated their awareness of the target (0 = no experience, 1 = brief experience, 2 = almost clear experience, 3 = clear experience). Participants used the rating as instructed, evidenced by a high proportion of 0 ratings for target-absent trials (i.e., contrast = 0; 76.88% ± 3.38%; see OSF Results for details). Because most ratings were either 0 (60.53% ± 4.05%) or 1 (32.73% ± 3.27%) in target-present trials, we divided trials into two types: unseen, wherein participants used a rating of 0, and seen, wherein participants used a rating of 1, 2, or 3. Feedback then showed target–probe discrimination accuracy (correct or incorrect) during the practice but not in the main experiment. (*B*) Occipital neuromodulation protocol (*Upper*) and current-flow model on three-dimensional reconstructions of the cortical surface (*Lower*). Details include the location and current intensity of each electrode. (*C*) Mean target–probe discrimination accuracy of target-present trials for unseen and seen trials before (baseline), during (online), and after (offline) modulation, as a function of frequency (see OSF Table S1 for accuracy and *A*′ values). Relative to baseline, β-modulation enhanced online and offline performance for seen trials; α-modulation enhanced offline performance for unseen trials. In contrast, β-modulation did not affect performance for unseen trials, and α-modulation did not affect performance for seen trials. θ-Modulation and sham did not affect performance for unseen or seen trials (all *Ps* > 0.05; see OSF Results for details). All multiple comparisons were Bonferroni-corrected. The frequency values were 20 Hz for β, 11 Hz for α, and 6 Hz for θ. Dashed gray lines are chance-level performance (50% correct). Circles are group means. Shaded error bars represent 1 SEM.

## Results

To determine the spectral properties governing conscious and unconscious visual processing, we used 2 × 4 HD-tACS to entrain nonharmonic β, α, or θ activity in bilateral occipital regions, guided by electrical field modeling (Fig. 1*B*; see *SI Appendix* for a brief discussion of tACS action mechanisms and the choice of frequencies). Participants performed a 30-min session of the task before (baseline), during (online), and after (offline) neuromodulation. The task comprised a brief (17 ms), masked Gabor target, followed by a Gabor probe appearing after a 1-s delay (Fig. 1*A*; see *SI Appendix* for detailed methods). Participants compared the orientations of the masked target and visible probe. The target–probe discrimination was followed by an awareness rating regarding the perception of the target. We analyzed target–probe discrimination performance as a function of awareness rating, and performed signal detection analyses to examine target awareness. We divided trials into two types: “unseen,” representing no awareness of the target, and “seen,” representing some awareness of the target (Fig. 1*A*).

Baseline accuracy for the target–probe discrimination was at chance-level for unseen trials (Fig. 1*C*) [β: *t*(17) = −0.49, *P* = 0.685; α: *t*(17) = 0.79, *P* = 0.220; θ: *t*(17) = 0.24, *P* = 0.406; sham: *t*(17) = −0.36, *P* = 0.637]. This confirms the effectiveness of the mask in precluding participants from using any target-related signal to guide the subsequent target–probe discrimination decision. As expected, baseline target–probe discrimination accuracy was above-chance for seen trials (Fig. 1*C*) [β: *t*(17) = 4.30, *P* < 0.001; α: *t*(17) = 2.92, *P* = 0.006; θ: *t*(17) = 2.31, *P* = 0.020; sham: *t*(17) = 4.69, *P* < 0.001].

Strikingly, HD-tACS improved conscious and unconscious processing in a sustainable and frequency-specific fashion (Fig. 1*C*). (We present the full results on the Open Science Framework [OSF] at https://osf.io/fqxk9/.) β-Modulation preferentially enhanced accuracy and sensitivity for target–probe discrimination for seen trials, but not unseen trials. The improvement started to manifest during neuromodulation (online) (accuracy: *P_Bonferroni_* = 0.001; sensitivity *A*′*: P_Bonferroni_* = 0.133), but became larger and more robust after neuromodulation (offline) (accuracy: *P_Bonferroni_* < 0.001; *A*′*: P_Bonferroni_* = 0.024), relative to baseline. In contrast, α-modulation enhanced accuracy and sensitivity for unseen trials, but not seen trials. The effect was significant offline (accuracy: *P_Bonferroni_* = 0.025; *A*′*: P_Bonferroni_* = 0.012) and only marginal online (accuracy: *P_Bonferroni_* = 0.071; *A*′*: P_Bonferroni_* = 0.078), relative to baseline. This larger and more robust offline benefit in β and α groups was likely due to the sluggish temporal application of transcranial electrical stimulation methods, which can lead to stronger behavioral effects after modulation (e.g., refs. 12 and 13). Additionally, neither θ nor sham groups showed any effects, lending further evidence for frequency specificity. In sum, the rhythmic mechanisms for improving conscious and unconscious visual perception appear dissociable along separate frequency “channels” of neural information processing and capable of being casually manipulated in a sustainable manner.

Signal detection analyses revealed that β-modulation selectively enhanced delayed target–probe discrimination accuracy without affecting target detection. The β group had above-chance detection sensitivity on seen trials in all sessions (0.70 < *A*′ < 0.75) (see OSF Table S2 for target detection sensitivity in all groups). The above-chance sensitivity means higher ratings when the target was present than absent, suggesting that participants used the rating scale appropriately. Importantly, β-modulation did not change: 1) detection sensitivity before, during, and after modulation [*F*(2, 51) = 1.46, *P* = 0.241]; and 2) the likelihood of reporting seeing the target [*F*(2, 51) = 0.21, *P* = 0.813; see OSF Results for decision criterion analyses]. In other words, β-modulation did not affect the amount of perceived target information. Instead, β-modulation improved the delayed target–probe discrimination for seen trials, reflecting selective tuning of conscious orientation information.

## Discussion

Our results demonstrate a dissociation of β- and α-rhythms in modulating the processing of seen and unseen information. This is causal evidence suggesting that β-rhythms facilitate short-term memory for visual information in conscious awareness (10). We propose that this results from β-rhythms enhancing the attentional mechanisms operating on visual short-term memory representations within the conscious domain. Since β-modulation did not boost perception of the target or the likelihood of reporting it, β-rhythms likely supported downstream processing, fine-tuning task-relevant information held in short-term memory, rather than supporting the initial attentional modulation that gates information into consciousness proposed by the global neuronal workspace theory (GNWT) (1) or the attended intermediate-level representations theory (14). On the other hand, α-modulation facilitated the processing of nonconscious information, presumably because it was task-relevant and in the focus of attention. This is distinct from the processing of task-irrelevant information at unattended locations that may be suppressed by spontaneous α-rhythms (15). Together, our findings suggest that β- and α-rhythms distinctly modulate conscious and unconscious information processing, respectively.

These findings have critical implications for neurobiological theories of consciousness (2). For example, the GNWT emphasizes the importance of β- and γ-synchronization for information processing in the global “workspace,” supporting conscious access (5). This is consistent with our finding that β-modulation enhances task-relevant information for conscious short-term memory processing, although we attribute the β-effect to downstream mechanisms. In contrast, the benefit of α-rhythms in unconscious processing is not predicted by any neurobiological theory of consciousness. Recent theoretical models suggest that unconscious information is silently maintained in synaptic weights in the absence of persistent neural firing (6, 16). Our demonstration of the causal role of α-rhythms in unconscious visual processing argues against this view. Stronger α leaves smaller temporal windows during processing, which can result in more precise excitatory (e.g., γ) processing (17), thereby boosting the global availability of unconscious contents (18) for short-term memory and decision-related mechanisms. Our study sets a framework for investigating the large-scale rhythmic mechanisms that generate conscious and unconscious experiences.

## Materials and Methods

Seventy-two healthy young participants (29 men, mean age: 20.56 y ± 0.41) (see *SI Appendix* for participant information within groups) consented to procedures approved by the Boston University Institutional Review Board and were paid. This study is between-participants and sham-controlled: we randomly assigned participants to one of four groups (β, α, θ, and sham; 18 participants per group). The procedure was identical across groups except for the HD-tACS protocol (*SI Appendix*).

## Supplementary Material

Supplementary File

## Data Availability

Data have been deposited in the Open Science Framework, https://osf.io/fqxk9/ (19).

## Footnotes

^*^The definition of consciousness is vigorously debated. Here, we use consciousness to mean availability to verbal report (1). Since we presented visual stimuli to participants, the stimuli had gained access to consciousness if they were reported seen, and not gained access if reported unseen. Therefore, “seen” and “conscious,” as well as “unseen” and “unconscious,” are used interchangeably.
