# Supplementary material for: Dissociable rhythmic mechanisms enhance memory for conscious and nonconscious perceptual contents
Source: Proc Natl Acad Sci U S A. 2022 Oct 27;119(44):e2211147119. doi: 10.1073/pnas.2211147119 (PMC9636912; doi:10.1073/pnas.2211147119)
Supplement: Supplementary File [file pnas.2211147119.sapp.pdf]

Dissociable rhythmic mechanisms enhance memory for conscious and nonconscious perceptual contents

Phillip (Xin) Cheng, Shrey Grover, Wen Wen, Shruthi Sankaranarayanan, Sierra Davies, Justine Fragetta, David Soto, Robert M. G. Reinhart

Supplementary Information

Methods

Participants

Seventy-two healthy young participants (29 men, mean age: 20.56 years ± 0.41; beta: n = 18, 7 men, mean age: 22.17 years ± 1.32; alpha: n = 18, 8 men, mean age: 19.17 years ± 0.26; theta: n = 18, 7 men, mean age: 19.89 years ± 0.50; sham: n = 18, 7 men, mean age: 20.39 years ± 0.41; age difference: $F(3, 68) = 2.55$, $p = .063$) took part in the study in exchange for $15 / hour. All participants reported normal or corrected-to-normal vision, normal color vision, no mental implants in the head, no implanted electronic devices, no history of neurological disorders or head injury, no skin sensitivity, not claustrophobic, not being pregnant and never being diagnosed with a substance problem. This study was approved by the Boston University Institutional Review Board.

Apparatus

Participants sat approximately 93 cm from a 24-in. monitor (resolution: 1,920 × 1,080 pixels; refresh rate: 144 Hz). MATLAB (MathWorks, Natick, MA) Psychophysics Toolbox Version 3 extensions (1) presented stimuli. A pair of photodiodes ensured the precise duration of stimulus presentation.

High-definition transcranial alternating current stimulation (HD-tACS). tACS non-invasively modulates macroscopic brain network dynamics by delivering low-intensity, sinusoidal electrical currents on the scalp (2). During tACS, the applied rhythmic electrical field gradually phase-locks the spiking activity of the targeted region (3). Consequently, the applied frequency entrains the intrinsic frequency of neuronal populations (4). The effect of tACS can last beyond the modulation period due to the strengthening of synaptic weights resulting from spike timing-dependent plasticity (5). HD-tACS takes advantage of the electrical field modeling to achieve maximized intensity or focality of modulation at the targeted region (6).

Here, a nine-channel high-definition transcranial electrical-current stimulator (Soterix Medical) delivered non-invasive alternating current. We used electrical field modeling (HD-Targets and HD-Explore developed by Soterix Medical) to guide the targeting of bilateral occipital regions. Electrical field modeling helped achieve more precise targeting and focal current distributions (7). The resultant montage comprised six sintered 12-mm-diameter Ag-AgCl electrodes placed in high-definition plastic holders, filled with conductive gel, embedded in an elastic HD-tACS cap (Soterix Medical). The model-guided location of electrodes and their current intensity values were: O1: 0.80 mA; O2: 0.80 mA; FC3: -1.04 mA; FC4: -0.29 mA; F8: -0.13 mA, FT10: -0.14 mA. The alternating current was bipolar sinusoidal with a peak-to-baseline intensity of 1.6 mA. The frequencies of the current were beta (20 Hz), alpha (11 Hz), or theta (6 Hz). The selection of frequency bands was based on time-frequency analysis in previous MEG research showing strong posterior activity from about 4 to 20 Hz when processing orientation information (8), which is key to our task (see Design and procedure). The frequency values avoided harmonics. Modulation lasted for 30 mins, simultaneous with the second session of the task (**Fig. 1a**; also see Design and procedure for details). In addition to the three active modulation groups, we included a passive sham group.

Having an identical procedure to the active modulation groups, the current for the sham group ramped up and down for only 30 s at the start and end of the 30-min period to simulate the sensation of active modulation (9). All participants reported warm, tingling skin sensations during modulation, and confirmed that the sensations were acceptable. Participants also reported weak flickering phosphenes in peripheral visual fields, but they confirmed that the phosphenes did not interfere with optimal task performance. At the end of the study, we administered a safety questionnaire (10) and a visual analog scale (11), which included questions regarding attention, concentration, mood, vision, headache, fatigue, and skin sensations under the modulating electrodes. Scores on these ratings did not significantly differ among groups ($Fs(3, 68) < 1.22$, $ps > .309$).

Stimuli

The target and probe stimuli (**Fig. 1a**) were Gabor gratings with a spatial frequency (SF) of 0.10, 0.11, or 0.12 cycles per pixel (c/p), centrally presented on a gray background (RGB value: 128, 128, 128). The mask consisted of radial square waves (spatial frequency = 0.11 cycles/pixel). The contrast was 0, 0.25, 0.75, or 1 with equiprobability for the target, 1 for the probe, and 1 for the mask. The target and probe had a diameter subtending 4.3° of visual angle, and the mask had a diameter of 6.8°. The target had six possible orientations: 10°, 40°, 70°, 100°, 130°, or 160° (measured clockwise from vertical), randomly occurring for any given trial. The orientation of the probe was always 30° different from the target, either clockwise or counterclockwise.

Design and procedure

Each trial began with a central fixation cross for 500 ms, followed by a presentation of the target for 17 ms (**Fig. 1a**). The 17 ms was the average duration of on-screen presentation measured by photodiodes, achieved by 13-ms presentation time in Psychophysics Toolbox. The SF of the target grating was either 0.10 or 0.12 c/p. 50 ms after the target, the mask appeared for 117 ms. A blank delay of 1000 ms followed, after which the probe stimulus appeared. The SF of this probe grating was 0.11 c/p. Participants pressed a key to indicate whether the target needed to rotate clockwise or anti-clockwise to match the orientation of the probe. The probe remained visible until participants made their target-probe discrimination response. Participants then rated their subjective awareness of the target on a 4-point Likert scale (0: no experience; 1: brief experience; 2: almost clear experience; 3: clear experience) (12). In practice trials, participants received on-screen feedback stating their target-probe discrimination accuracy after giving their awareness rating. There was no feedback in the main phase of the experiment. The intertrial interval was 500 ms.

We randomly assigned participants to one of four groups (beta, alpha, theta, and sham). The procedure was identical across groups except for the HD-tACS protocol (see Apparatus). The task contained three sessions, one before modulation (baseline), one during modulation (online), and one after modulation (offline). Each session had three blocks. Within each block, 5 randomly located catch trials had a full-contrast target presented for 500 ms. The target was clearly visible in catch trials, allowing us to only include participants who performed the task as instructed.

Each participant performed 20 practice trials before the baseline session. The first 10 trials of practice were catch trials to help participants understand the task. In the second 10 trials of practice, the target appeared for 17 ms, identical to the main experiment. There were 864 trials in the main experiment, 288 trials in each session, with 3 repetitions of each combination of target SF (0.10/0.12 c/p), target contrast (0, 0.25, 0.75 and 1), target orientation (10-160° in 6 steps) and probe tilt (clockwise/anti-clockwise), in random order. Participants took a self-paced break after every 96 trials.

Participants were instructed to (i) maintain their focus throughout all the sessions; (ii) try their best to detect the brief target; and (iii) accurately rate their awareness of the target; in particular, use the zero rating only if they felt they had not seen the target at all.

The masking paradigm differs from previous versions in two main aspects, rendering the current version novel. First, the target was set to appear for 13 ms in Psychophysics Toolbox, shorter than the 17 ms in previous studies (8, 13, 14). Second, the mask comprised radial square waves, distinct from an energy mask (13, 14), sine wave mask (8), and pattern mask (15) used in previous consciousness studies.

Data analysis

All participants achieved a greater than 60% accuracy in catch trials (mean accuracy: 81.98% ± 0.02%), so all were included for further analysis. Trials did not have timeout and the task instruction emphasized accuracy, consequently our analysis focused on discrimination accuracy across awareness ratings.

We analyzed the target-probe discrimination performance in terms of accuracy and sensitivity (*A'*) using target-present trials (contrast = 0.25, 0.75, or 1), as a function of participants' self-reported awareness of the target on each trial, with levels of target contrast collapsed as well as separated. *A'* is suitable as the sensitivity measure for target-probe discrimination because it (i) is free from response bias (6), and (ii) prevents distortion of the measure of performance at the group level when the signal is weak (16).

$$A' = .5 + \left[sign(H - F) \frac{(H-F)^2 + |H-F|}{4max(H,F) - 4HF} \right]$$

H is the hit rate; F is the false alarm rate. 'Hit' was when the probe stimulus was rotated clockwise compared to the target and a clockwise response was given. 'Miss' was when the rotation was clockwise and a counterclockwise response was given. 'False alarm' was when the rotation was counterclockwise, but a clockwise response was given. 'Correct rejection' was when both the rotation and response were counterclockwise. A hit rate is the ratio between 'Hit' and the sum of 'Hit' and 'Miss'. A false alarm rate is the ratio between 'False alarm' and the sum of 'False alarm' and 'Correct rejection'. The computation of *A'* used individual rates of hits and false alarms, a standard procedure based on the signal detection theory (17). We compared overall performance (i) with the chance level; (ii) across frequencies of modulation; and (iii) across sessions within a modulation frequency. We present discrimination accuracy and *A'* for all conditions in **Table S1**. Because most ratings were either '0' (60.53% ± 4.05%) or '1' (32.73% ±

3.27%), we divided trials into two types: those wherein participants indicated no awareness of the target (awareness rating of 0) or ‘unseen’ trials, and those wherein participants indicated some awareness of the target (trials with awareness rating of 1, 2 or 3, combined) or ‘seen’ trials. We excluded participants’ responses from a session if they had fewer than 10 unseen or seen trials in this session to ensure a reliable estimate of performance. Accuracy was significantly below chance in one participant. This means that the participant systematically misremembered the relationship between the specific target-probe rotation and the corresponding response key. Because the probe was clearly visible until a response was made, systematic misremembering means that they were able to perform the delayed target-probe discrimination but failed to use the relevant response mapping (18). Accordingly, we reversed participants’ target-probe discrimination responses if their accuracy was lower than 40% (19).

In addition, we performed signal detection analyses to examine perceptual awareness of the target. The purpose was to test (i) whether target contrast affected target detection; and (ii) whether neuromodulation affected target detection in general or specifically influenced target-probe orientation discrimination. We calculated sensitivity *A’*. We present sensitivity for all four groups in **Table S2** on the Open Science Framework (https://osf.io/fqxk9/). ‘Hit’ was when the target was present and reported seen (awareness rating = 1, 2, or 3). ‘False alarm’ was when the target was absent and reported seen. Note that detection sensitivity does not apply to trials where participants reported not seeing the target (awareness rating = 0). Previous attempts to redefine ‘Hit’ and ‘False alarm’ have been shown to provide a ‘pseudo-sensitivity’’ (13, 14), a biased measure of detection sensitivity varying with response criterion (20).

Analyses were two-tailed unless stated otherwise. We used generalized linear mixed-effect models (GLMM) to analyze accuracy across and within tACS modulation groups. The GLMM offers higher power than conventional statistical tests (21) and is ideal for tasks involving subjective ratings that inevitably end up with unbalanced data (22). For these analyses, we specified effects as random at the participant level and reported them at the population level. To control for Type I errors, we corrected multiple comparisons using the Bonferroni procedure.

References

1. M. Kleiner, D. Brainard, D. Pelli, ECVP '07 abstracts. *Perception* **36**, 1–235 (2007).
2. D. J. L. G. Schutter, Syncing your brain: electric currents to enhance cognition. *Trends Cogn. Sci.* **18**, 331–333 (2014).
3. M. R. Krause, P. G. Vieira, B. A. Csorba, P. K. Pilly, C. C. Pack, Transcranial alternating current stimulation entrains single-neuron activity in the primate brain. *Proceedings of the National Academy of Sciences* **116**, 5747–5755 (2019).
4. R. F. Helfrich, *et al.*, Entrainment of Brain Oscillations by Transcranial Alternating Current Stimulation. *Curr. Biol.* **24**, 333–339 (2014).
5. T. Zaehle, S. Rach, C. S. Herrmann, Transcranial alternating current stimulation enhances individual alpha activity in human EEG. *PLoS One* **5**, e13766 (2010).
6. S. Grover, J. A. Nguyen, R. M. G. Reinhart, Synchronizing Brain Rhythms to Improve Cognition. *Annu. Rev. Med.* **72**, 29–43 (2021).
7. D. Edwards, *et al.*, Physiological and modeling evidence for focal transcranial electrical brain stimulation in humans: a basis for high-definition tDCS. *Neuroimage* **74**, 266–275 (2013).
8. J.-R. King, N. Pescetelli, S. Dehaene, Brain Mechanisms Underlying the Brief Maintenance of Seen and Unseen Sensory Information. *Neuron* **92**, 1122–1134 (2016).
9. R. M. G. Reinhart, J. D. Cosman, K. Fukuda, G. F. Woodman, Using transcranial direct-current stimulation (tDCS) to understand cognitive processing. *Atten. Percept. Psychophys.* **79**, 3–23 (2017).
10. C. Poreisz, K. Boros, A. Antal, W. Paulus, Safety aspects of transcranial direct current stimulation concerning healthy subjects and patients. *Brain Res. Bull.* **72**, 208–214 (2007).
11. P. C. Gandiga, F. C. Hummel, L. G. Cohen, Transcranial DC stimulation (tDCS): a tool for double-blind sham-controlled clinical studies in brain stimulation. *Clin. Neurophysiol.* **117**, 845–850 (2006).
12. M. Overgaard, B. Timmermans, K. Sandberg, A. Cleeremans, Optimizing subjective measures of consciousness. *Conscious. Cogn.* **19**, 682–4; discussion 685–6 (2010).
13. D. Soto, T. Mäntylä, J. Silvanto, Working memory without consciousness. *Curr. Biol.* **21**, R912–R913 (2011).
14. A. Dutta, K. Shah, J. Silvanto, D. Soto, Neural basis of non-conscious visual working memory. *Neuroimage* **91**, 336–343 (2014).
15. P. X. Cheng, A. N. Rich, M. E. Le Pelley, Reward Rapidly Enhances Visual Perception. *Psychol. Sci.* **32**, 1994–2004 (2021).
16. H. Stanislaw, N. Todorov, Calculation of signal detection theory measures. *Behav. Res. Methods Instrum. Comput.* **31**, 137–149 (1999).

17. T. D. Wickens, *Elementary Signal Detection Theory* (Oxford University Press, 2001).

18. S. K. Robins, Misremembering. *Philos. Psychol.* **29**, 432–447 (2016).

19. R. I. Frederick, F. M. Speed, On the interpretation of below-chance responding in forced-choice tests. *Assessment* **14**, 3–11 (2007).

20. T. Stein, D. Kaiser, G. Hesselmann, Can working memory be non-conscious? *Neurosci Conscious* **2016**, niv011 (2016).

21. R. H. Baayen, *Analyzing Linguistic Data* (Cambridge University Press, 2008) (May 29, 2022).

22. C. Muth, *et al.*, Alternative Models for Small Samples in Psychological Research: Applying Linear Mixed Effects Models and Generalized Estimating Equations to Repeated Measures Data. *Educ. Psychol. Meas.* **76**, 64–87 (2016).
